# Acknowledgement to Reviewers of *Biomedicines* in 2014

**DOI:** 10.3390/biomedicines3010045

**Published:** 2015-01-09

**Authors:** Biomedicines Editorial Office

**Affiliations:** MDPI AG, Klybeckstrasse 64, CH-4057 Basel, Switzerland

The editors of *Biomedicines* would like to express their sincere gratitude to the following reviewers for assessing manuscripts in 2014:

Ali, Robin R.
Allgayer, Heike
Beck, Alain
Bottaro, Donald P.
Boyd, Chris
Cara, Andrea
Castro, Maria
Chade, Alejandro R.
Choi, Woonyoung
Crepaldi, Tiziana
Dingermann, Theodor
Dotti, Gianpietro
Egloff, Ann Marie
Ferlini, Cristiano
Flashman, Emily
Fueyo, Juan
Gabbay, Kenneth H.
Gallick, Gary E.
Grimm, Elizabeth A.
Hunsberger, Joshua G.
Jenkins, Brendan J.
Kao, Chinghi
Koeberl, Dwight D.
Landau, Nathaniel
Lieber, Andre
Matsumoto, Kunio
McGuckin, Colin
Medová, Michaela
Micdavis, Hael E.
Montini, Eugenio
Morral, Nuria
Nakamura, Toshikazu
Nejak-Bowen, Kari N.
Nicastro, Holly L.
Niemann, Hartmut H.
Noonan, Frances P.
Ouchi, Toru
Papaccio, Gianpaolo
Park, Morag
Pérez-Tomás, Ricardo Enrique
Philips, Neena
Porras, Almudena
Rabinowitz, Joseph E.
Raina, Komal
Ricci, Giulia
Scarlatti, Gabriella
Schneider, Ian C.
Seymour, Len W.
Shojaei, Farbod
Smerdou, Christian
Takagi, Hitoshi
Tamagnone, Luca
Thorne, Steve
Timpson, Paul
Torigoe, Toshihiko
Van Dongen, Guus A. M. S.
Vermeulen, Louis
Von Kalle, Christof
Yáñez-Muñoz, Rafael J.
Zachow, Rob
Zentilin, Lorena

